# The Preference for Joint Attributions Over Contrast-Factor Attributions in Causal Contrast Situations

**DOI:** 10.3389/fpsyg.2019.01881

**Published:** 2019-08-23

**Authors:** Moyun Wang, Mingyi Zhu

**Affiliations:** Shaanxi Key Laboratory of Behavior and Cognitive Neuroscience, School of Psychology, Shaanxi Normal University, Xi’an, China

**Keywords:** causal attribution, contrast situation, mechanism, joint attribution, explanatory complexity, explanatory sufficiency

## Abstract

A current issue about causal attribution is whether people take simple contrast-factor attributions or complex joint attributions in contrast situations. For example, a stone does not dissolve in water and a piece of salt dissolves in water. That the piece of salt dissolves in water is due to: (A) the influence of the piece of salt; (B) the influence of the water; (C) the joint influence of the piece of salt and the water. We propose a mechanism-based sufficiency account for such questions. It argues that causal attributions are guided by mechanism-based explanatory sufficiency, and people prefer a mechanism-based attribution with explanatory sufficiency. This account predicts the sufficient joint attribution (the C option), whereas the conventional covariation approach predicts the contrast-factor attribution (the A option). Two experiments investigated whether contrast situations affect causal attributions for compound causation with explicit mechanism information and simple causation without explicit mechanism information, respectively. Both experiments found that in both the presence and absence of contrast situations, the majority of participants preferred sufficient joint attributions to simple contrast-factor attributions regardless of whether explicit mechanism information was present, and contrast situations did not affect causal attributions. These findings favor the mechanism-based sufficiency account rather than the covariation approach and the complexity account. In contrast situations, the predominance of joint attributions implies that explanatory complexity affects causal attributions by the modulation of explanatory sufficiency, and people prefer mechanism-based joint attributions that provide sufficient explanations for effects. The present findings are beyond the existing approaches to causal attributions.

## Introduction

In everyday life (social and physical scenarios), people often need to make causal attributions in contrast situations. For example, when Jim criticizes Fisher, Fisher does not get angry, and when Gibson criticizes Fisher, Fisher becomes angry. For this social scenario, a question is to infer which of three options (Fisher’s influence, Gibson’s influence, Fisher and Gibson’s joint influence) lead to that Fisher becomes angry. A physical scenario is as follows: a piece of coal sinks in water, and a piece of wood floats on water (Bender and Beller, 2011; Beller and Bender, 2015). For this situation, a question is to infer what causes the piece of wood to float. This situation involves the contrast between two different causal instances. The fact that a piece of wood floats on water is the foreground causation in which the piece of wood floating needs to be explained, whereas the fact that a piece of coal sinks in water is the background causation that does not need to be explained. The example questions can vary with two response formats: two versus three options. For example, the wood question can have two response options: (1) the piece of wood, (2) the water, or three response options: (1) the piece of wood, (2) the water, (3) the joint influence of the piece of wood and the water. A current issue about such questions is whether people take simple contrast-factor attributions or complex joint attributions in causal contrast situations. No previous studies have investigated this issue. Therefore, it is noteworthy to clarify this issue.

In contrast situations, there are two main approaches to causal attributions: the covariation and mechanism approaches (White, 1989, 2009, 2012, 2014; Morris and Peng, 1994; Buehner, 2005; Danks, 2005; Alicke et al., 2015; Hilton, 2017; Johnson and Ahn, 2017). A basic concern is about contrast situations involving singular contrast factors. For such situations, the covariation approach predicts simple contrast-factor attributions, whereas the mechanism approach predicts complex joint attributions. We specify the two predictions as follows.

The covariation approach assumes that people compare different situations to find what factors make a difference to an effect, and attribute the effect to these factors (Heider, 1958; Kelley, 1973; Cheng and Novick, 1990, 1992; McGill and Klein, 1993; Cheng, 1997; Buehner, 2005; Perales and Shanks, 2007; Bender and Beller, 2011; Alicke et al., 2015; Beller and Bender, 2015; Hilton, 2017). If two situations show only one difference-making factor, it will be regarded as causative. For the wood question, comparing the background and foreground situation suggests the covariation between the agents (the coal versus the wood) and the effects (sinks in water versus floats on water). The covariation predicts the contrast-factor attribution that the wood floating is due to the wood. The covariation approach implies simple causal explanations for contrast situations involving singular contrast factors.

The mechanism approach treats causal attribution as a search for underlying mechanisms, and assumes that mechanism knowledge guides and constrains causal attributions, and people are sensitive to causal mechanism information (White, 1989, 2009, 2012, 2014; Ahn and Bailenson, 1996; Fugelsang and Thompson, 2000; Johnson and Ahn, 2017; Lake et al., 2017; Bramley et al., 2018). A causal mechanism is generally defined as a system of entities, parts, or variables that causally interact in organized or systematically predictable ways to result in a phenomenon or an effect (Machamer et al., 2000; Illari and Williamson, 2012; Craver and Tabery, 2015; Johnson and Ahn, 2017). The original mechanism approach predicts only that when covariation information and mechanism knowledge predict different single-factor attributions, the influence of mechanism knowledge will trump the influence of covariation information such that people will prefer mechanism-based to covariation-based attributions (Ahn et al., 1995; Fugelsang and Thompson, 2000). According to the mechanism approach, we further think that an effect is more likely to be brought about by the joint influence of multiple relevant factors in the causal mechanism, rather than one of these factors. Accordingly, people may attribute the effect to the joint influence of multiple relevant factors, showing the mechanism-based joint attribution. We for the first time clarify this explicit prediction, which is absent in the original mechanism approach. The mechanism approach predicts complex joint attributions for the wood question. According to floating mechanics, the wood floating results from the resultant force of the weight of the wood and the buoyancy of the water, independent of whether the background causation is present. Thus, the wood floating is due to the joint influence of the wood and the water.

In many cases, causal attributions depend on causal explanations in that a good causal attribution should provide a good explanation. Previous studies on causal explanation suggest that explanatory factors such as explanatory sufficiency and simplicity may affect people’s preferences for causal explanations (Glymour, 2001; Johnson and Ahn, 2017; Lombrozo and Vasilyeva, 2017; Zemla et al., 2017). Thus, we conjecture that the two approaches to causal attributions may be associated with some explanatory factors. For the wood question, the covariation approach implies an insufficient and simple explanation, whereas the mechanism approach implies a more sufficient and complex explanation. Here, explanatory sufficiency and simplicity predict opposite causal attributions. Explanatory sufficiency predicts preferring the sufficient and complex joint attribution, whereas explanatory simplicity predicts preferring the insufficient and simple contrast-factor attribution.

For the above issue, we think that the influence of explanatory sufficiency will trump the influence of explanatory simplicity, because a good causal attribution should primarily be sufficient for the effect. This prediction is suggested by the recent finding that for a causal explanation question without contrasts, people prefer complex explanations invoking two independent causes to simple explanations invoking one of the two causes because the former are more sufficient than the latter (Zemla et al., 2017). They proposed a complexity account that people prefer complex explanations because complex explanations are more sufficient than simple explanations. However, this account does not emphasize the role of mechanisms.

We propose a mechanism-based sufficiency account for causal attributions. It integrates mechanisms and explanatory sufficiency. Its basic idea is as follows. Causal attributions are guided by mechanism-based explanatory sufficiency, and people will prefer a causal attribution based on mechanism knowledge if it is more sufficient for the effect than other alternative attributions. For a contrast situation with only one contrast factor, the mechanism-based sufficiency account predicts that whether people take the joint attribution based on mechanism knowledge or the contrast-factor attribution based on covariation information may depend on explanatory sufficiency. The mechanism-based joint attribution is more sufficient than the contrast-factor attribution. Thus, people should prefer the former to the latter. Here, mechanism knowledge may be concrete and explicit, or abstract and implicit. When no explicit concrete mechanism knowledge is available, people may appeal to implicit abstract mechanism knowledge, which is like intuitive theories proposed by Gerstenberg and Tenenbaum (2017). For example, people may know only that an interaction between two objects results in that one of the two objects breaks, but do not know the concrete mechanism of how the interaction works in detail.

It is noteworthy that the mechanism-based sufficiency account differs from the conventional mechanism approach. The former emphasizes the role of explanatory sufficiency, which is neglected by the latter. Moreover, the mechanism-based sufficiency account also differs from the complexity account. The former argues that causal attributions are directly related to explanatory sufficiency based on mechanisms, whereas the latter emphasizes the role of explanatory complexity, but neglects the role of mechanisms. When explanatory sufficiency and complexity predict opposite explanations, simpler but sufficient explanations versus more complex but over-sufficient explanations, the mechanism-based sufficiency account predicts preferring the former, whereas the complex account predicts preferring the latter.

Previous studies on causal attributions neglected investigating the role of explanatory factors in contrast situations. And previous studies on explanation focused on causal situations without contrasts, but neglected investigating contrast situations. To our knowledge, no previous studies have investigated the role of explanatory sufficiency and simplicity in causal attribution in contrast situations. Present research also investigates this important theoretical issue.

Beller et al. examined whether contrast situations affect causal attributions by using the force-choice format with two options: a constant factor and a contrast factor (Beller et al., 2009; Bender and Beller, 2011; Beller and Bender, 2015). These studies found that for the wood question, the majority of participants responded “it is due to the piece of wood,” showing the contrast-factor attribution and the contrast effect. The contrast-factor attribution is biased because it deviates from the physically sufficient explanation that the piece of wood floating is due to the joint force of the piece of wood and the water regardless of whether there is the background causation.

Why do people show such biases? There are two possible reasons. One may be the demand effect that the force-choice format forced people to choose one of two options, and thereby solicits preferring contrast factors over constant factors. The other possible reason lies in the role of contrast situations. In the wood question, the contrast between the background and foreground set the covariation between the two agents and the two effects. The covariation may induce preferring the contrast factor over the constant factor in the foreground causation, as is predicted by the covariation approach.

We think that the force-choice dichotomous response format may be the primary reason of the bias. Beller and Bender (2015) examined causal assignments and explanations for CO_2_ and air in the contrast situation problem where helium rises in air whereas CO_2_ stays down in air. Their task asked participants to first make quantitative ratings for the relative contributions of CO_2_ and air to the fact that CO_2_ stays down in air, and then give causal explanations for the fact. They found that the participants gave more weight for the contrast factor CO_2_ than for the constant factor air, but the majority (53%) of the participants gave the relational explanation that the fact is due to the interaction of CO_2_ and air. This result suggests that in contrast situations, people can make mechanism-based complex explanations, and thereby contrast situations are not bound to elicit contrast-factor attributions. Thus, the bias probably resulted from the force-choice dichotomous response format rather than the contrast situations. This response format rendered participants to fall into biased dichotomous choice without considering the sufficient joint explanation.

For the wood question, when using the three-option response format involving the joint option, the mechanism-based sufficiency account predicts that people will prefer the joint option to the two single-factor options, because the joint option is a sufficient explanation, whereas the other two options are insufficient explanations.

To our knowledge, no previous studies have systematically investigated whether people prefer complex joint attributions to simple contrast-factor attributions in contrast situations (Alicke et al., 2015; Hilton, 2017; Johnson and Ahn, 2017). We conducted two experiments to investigate the issue. Experiment 1 examined causal attribution in contrast situations with concrete mechanism information. Experiment 2 further examined causal attribution in contrast situations with no concrete mechanism information.

## Experiment 1

### Method

#### Participants

A total of 80 college students (35 males, 45 females) from Shaanxi Xueqian Normal University in China participated in Experiment 1. The study was approved by the Shaanxi Normal University Human Research Ethics Committee. All the participants provided written informed consent.

#### Design and Materials

Experiment 1 was a paper-and-pencil questionnaire study. We used a between-subjects design with situation (the absence versus presence of contrasts) as the between-subjects factor. There were two groups: the contrast group and the baseline group without contrasts. The contrast group received a contrast problem with a contrast situation comprising two causal instances: one was the foreground compound causation in which two agents jointly resulted in an effect, and the other was the background simple causation in which only one agent resulted in an effect. The two causal instances shared a common agent as the constant factor. One of the two agents in the foreground causation was the contrasting factor that was absent in the background causation. The contrast problem is presented as follows. Here is the English version translated from the original Chinese version.

**Instruction**

Please read the following contents and then tick one option as your answer to each question. Thank you for your cooperation.

**Figure 1:**
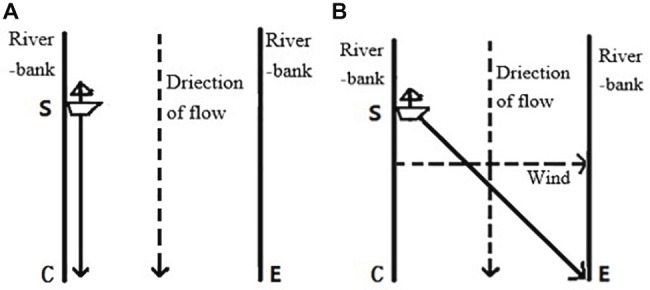
The contrast of two situations in Experiment 1.

1. In Figure 1A, an unmanned sailboat berthed at Location S is in a river at the beginning. Then it was affected by the current and eventually reached Location C. In Figure 1B, the sailboat berthed at Location S at the beginning. Then, a wind from the left to the right rose. The sailboat was affected by the current and the wind. Finally, it reached Location E. Compared with that the sailboat reached Location C in Figure 1A, that the sailboat reached Location E in Figure 1B was due to: (A) the influence of the current; (B) the influence of the wind; (C) influence of the sailboat; (D) the joint influence of the current and the wind; (E) the joint influence of the current, the wind, and the sailboat.

The baseline group received the baseline problem that was adapted from the contrast problem in the contrast group by removing the content of the background causation.

According to the mechanism-based sufficiency account, option D was the mechanism-based sufficiency attribution because the movement of the sailboat was determined by the joint force of the current and the wind, whereas each of the three single-factor options was too simple and thereby was insufficient for the arrival of the sailboat. Option E involved unnecessary explanatory information (that is, the sailboat) because the sailboat itself was not a cause of its movement. Thus, Option E was over-complex and over-sufficient so that it was not appropriate. Consequently, the mechanism-based sufficiency account predicted that participants would prefer option D to the other options regardless of whether the contrast situation was present. Conversely, the covariation approach predicted that participants would prefer contrast-factor attributions in the contrast situation. Moreover, according to the complexity account (Zemla et al., 2017), participants would prefer option E, which was the most complex attribution.

#### Procedure

The participants were assigned to one of the two groups. They took about 5 min to complete the questionnaires in quiet classrooms. Each participant received a pen for participation.

## Results and Discussion

The results are shown in Table 1. For each problem, the majority of participants (no less than 75%) judged that the effect is due to the joint force of the current and the wind, showing sufficient joint attributions. The proportion of the sufficient joint attributions was significantly higher than the overall proportion of the other attributions, which included contrast-factor attributions. For the baseline problem, *χ*^2^(1) = 12.1, *p* < 0.005, *W* = 0.6; for the contrast problem, *χ*^2^(1) = 10, *p* < 0.005; *W* = 0.5. Overall, participants preferred the sufficient joint attribution to the other attributions.

**Table 1:** The frequencies (percentages) of causal attributions in Experiment 1.

Group	Causal attributions				
	A Constant agents	B Contrast agents	C Patients	D Two agents	E Three factors
The baseline group	0 (0)	0 (0)	0 (0)	31 (77.5)	9 (22.5)
The contrast group	0 (0)	6 (15)	0 (0)	30 (75)	4 (10)

We conducted a 2 (situation: the contrast versus baseline group) × 2 (type of causal attributions: the sufficient attributions versus the other attributions) Chi-square independence test for the two problems. It showed no differences between the two groups, *χ*^2^(1) = 0.07, *p* > 0.05. Compared with the baseline group, the contrast group showed no contrast effects.

In summary, for compound causation with mechanism information, contrast situations did not affect causal attributions in contrast situations. In both the presence and absence of contrast situations, participants generally preferred mechanism-based sufficient joint attributions to the other attributions. This result is consistent with the prediction of the mechanism-based sufficiency account, but not the prediction of the covariation approach and the complexity account.

### Experiment 2

Experiment 1 found that for compound causation with mechanism information, sufficient joint attributions were predominant in contrast situations. Experiment 2 further investigated whether people can make joint attributions for simple causation without explicit concrete mechanism information in contrast situations. In order to test the contrast effects found in the previous studies (Beller et al., 2009; Bender and Beller, 2011; Beller and Bender, 2015), Experiment 2 used the contrast design that is similar to that in Beller and Bender (2015). Each problem gave a contrast situation that consisted of two causal statements without explicit mechanism information. Each causal statement involved a simple causation between an agent and a patient.

### Method

#### Participants

A total of 80 college students (36 males, 44 females) from Xi’an University of Finance and Economics in China participated in Experiment 2. The study was approved by the Shaanxi Normal University Human Research Ethics Committee. All the participants provided written informed consent.

#### Design and Materials

The experiment was a paper-and-pencil questionnaire study. We used a between-subjects design with situation (the absence versus presence of contrasts) as the between-subjects factor. There were two groups: the baseline and contrast group.

The contrast group received a questionnaire containing eight problems: four natural problems and four social problems. Each problem involved a contrast of two causal statements: one is the background causation and the other is the foreground causation. The former is followed by the latter. A question following the two statements asked participants to judge whether the effect in the foreground causation is due to the influence of the agent, the influence of the patient, or the joint influence of the agent and patient. There were two kinds of contrast problems: four agent-contrast problems and four patient-contrast problems. An agent-contrast problem involved the contrast between two agents in causation (e.g., a piece of tofu does not break a piece of glass. A stone breaks a piece of glass). A patient-contrast problem involved the contrast between two patients in causation (e.g., a stone does not break a steel plate. The stone breaks a piece of glass). The two kinds of contrasts were orthogonally combined with two setting types of causation: symmetrical and asymmetrical settings. In a symmetrical setting, the agent and patient play an equal role in causation such that it is difficult to discern which is active or passive. In an asymmetrical setting, the agent and patient play unequal roles in causation such that it is easy to discern which is active or passive. Two symmetrical instances were that the piece of salt dissolves in water and that Ben laughs when watching Carter’s performance. Two asymmetrical instances were that the stone breaks a piece of glass, and the instance that when Gibson criticizes Fisher, Fisher becomes angry.

The contrast group’s questionnaire is presented as follows. It is the English version translated from the original Chinese version. The problems in the questionnaire were counterbalanced.

#### Instruction

Please read the following contents and then tick one option as your answer to each question.

1. A piece of tofu does not break a piece of glass. A stone breaks the piece of glass. That the piece of glass is broken is due to: (A) the influence of the stone, (B) the influence of the piece of glass, (C) the joint influence of the stone and the piece of glass.
2. Ben does not laugh when watching Ethan’s performance. Ben laughs when watching Carter’s performance. That Ben laughs is due to: (A) Ben’s influence, (B) Carter’s influence, (C) Ben and Carter’s joint influence.
3. A stone does not dissolve in water. A piece of salt dissolves in water. That the piece of salt dissolves in water is due to: (A) the influence of the piece of salt, (B) the influence of the water, (C) the joint influence of the piece of salt and the water.
4. When Jim criticizes Fisher, Fisher does not get angry. When Gibson criticizes Fisher, Fisher becomes angry. That Fisher becomes angry is due to: (A) Fisher’s influence, (B) Gibson’s influence, (C) Fisher and Gibson’s joint influence.
5. A stone does not break a steel plate. The stone breaks a piece of glass. That the piece of glass is broken is due to: (A) the influence of the stone, (B) the influence of the piece of glass, (C) the joint influence of the stone and the piece of glass.
6. Duncan does not laugh when watching Carter’s performance. Ben laughs when watching Carter’s performance. That Ben laughs is due to: (A) Ben’s influence, (B) Carter’s influence, (C) Ben and Carter’s joint influence.
7. A piece of salt does not dissolve in alcohol. The piece of salt dissolves in water. That the piece of salt dissolves in water is due to: (A) the influence of the piece of salt, (B) the influence of the water, (C) the joint influence of the piece of salt and the water.
8. When Gibson criticizes Hugo, Hugo does not get angry. When Gibson criticizes Fisher, Fisher becomes angry. That Fisher becomes angry is due to: (A) Fisher’s influence, (B) Gibson’s influence, (C) Fisher and Gibson’s joint influence.

The baseline group’s questionnaire contained four problems that were adapted from the contrast group’s problems by removing the background causation information. The four problems involved the following four respective statements:

A stone breaks the piece of glass.
A piece of salt dissolves in water.
Ben laughs when watching Carter’s performance.
Fisher becomes angry when he is criticized by Gibson.

To avoid the possible influence of school education on participants’ specific mechanism knowledge, we did not use problems in which causal contents such as the wood floating are taught in school education. Instead, we used the problems in which causal contents are generally absent in school education.

The mechanism-based sufficiency account predicted that participants would prefer joint attributions to contrast-factor attributions because the former are more sufficient than the latter. Conversely, the covariation approach predicted that participants would prefer contrast-factor attributions.

#### Procedure

The procedure was identical to that in Experiment 1.

## Results and Discussion

The results are shown in Table 2. There were no significant differences between symmetrical and asymmetrical settings for causal relationships. Thus, the setting type of causation did not affect causal attributions.

**Table 2:** The frequencies (percentages) of causal attributions in Experiment 2.

Problems	The baseline group			The contrast group					
				Agent-contrast			Patient-contrast		
	Agent	Patient	Joint	Agent	Patient	Joint	Agent	Patient	Joint
Glass breaking	8 (20)	0 (0)	32 (80)	9 (22.5)	1 (2.5)	30 (75)	2 (5)	16 (40)	22 (55)
Dissolution	3 (7.5)	12 (30)	25 (62.5)	4 (10)	4 (10)	32 (80)	2 (5)	15 (37.5)	23 (57.5)
Laugh	2 (5)	4 (10)	34 (85)	6 (15)	11 (27.5)	23 (57.5)	5 (12.5)	7 (17.5)	28 (70)
Anger	4 (10)	8 (20)	28 (70)	6 (15)	10 (25)	24 (60)	0 (0)	7 (17.5)	33 (82.5)
Overall	17 (10.62)	24 (15.00)	119 (74.38)	25 (15.62)	26 (16.25)	109 (68.13)	9 (5.62)	45 (28.13)	106 (66.25)

For the baseline group, we tested the difference between joint and single-factor attributions for each problem. For the contrast group, we tested the difference between joint and contrast-factor attributions for each problem. The results of the Chi-square tests of uniform distributions are shown in Table 3.

**Table 3:** The results of the Chi-square tests of uniform distributions in Experiment 2.

Problems	The baseline group	The contrast group	
		Agent-contrast	Patient-contrast
Glass breaking	14.40***	11.31***	0.95
Dissolution	2.50	21.78***	1.68
Laugh	19.60***	9.97**	12.60***
Anger	6.40*	10.8**	16.9***
Overall	38.03***	21.03***	16.90***

*df = 1*.

* p < 0.05,

** p < 0.01,

*** *p < 0.001*.

In the baseline group, for three of the four problems, the proportions of joint attributions were significantly larger than the respective proportions of single-factor attributions. In the contrast group, for six of the eight problems, the proportions of joint attributions were significantly larger than the respective proportions of contrast-factor attributions. Although few problems showed no significant differences, the majority (more than or equal to 55%) of participants preferred joint attributions. The problems showing no significant differences may be due to that the sample of responses is not enough large. When the results were collapsed across problems, the overall results with the large sample of responses showed significant differences between joint attributions and contrast-factor attributions, as shown in Table 3. Overall, for each problem in both the baseline and contrast group, the majority of participants preferred joint attributions.

We conducted a 2 (situation: no-contrast versus agent-contrast) × 2 (type of attributions: single-factor versus joint attributions) Chi-square independence test for each pair of problems. For the problems in Table 2, from top to bottom, the test results are as follows: *χ*^2^(1) = 0.29, *p* > 0.05; *χ*^2^(1) = 2.99, *p* > 0.05; *χ*^2^(1) = 7.38, *p* < 0.05; *χ*^2^(1) = 0.88, *p* > 0.05. For the Laugh scenario, the two groups showed a significant difference. This was because the contrast question yielded less joint attributions although joint attributions were preferred by the majority. For the overall results, the agent-contrast and no-contrast condition showed no differences, *N* = 320, *χ*^2^(1) = 1.53, *p* > 0.05.

We also conducted a 2 (situation: no-contrast versus patient-contrast) × 2 (type of attributions: single-factor versus joint attributions) Chi-square independence test for each pair of problems. For the problems in Table 2, from top to bottom, the test results are as follows: *χ*^2^(1) = 5.70, *p* < 0.05; *χ*^2^(1) = 0.21, *p* > 0.05; *χ*^2^(1) = 2.58, *p* > 0.05; *χ*^2^(1) = 1.73, *p* > 0.05. For the glass breaking scenario, the two groups showed a significant difference. This was because the contrast question yielded less joint attributions although joint attributions were preferred by the majority. For the overall results, the patient-contrast and no-contrast condition showed no differences, *N* = 320, *χ*^2^(1) = 2.53, *p* > 0.05.

In summary, for simple causation without explicit mechanism information, the contrast group did not show more contrast-factor attributions than the baseline group, and thereby contrast situations did not affect causal attributions. In both the presence and absence of contrasts, participants generally preferred sufficient joint attributions to insufficient contrast-factor attributions. These results are consistent with the prediction of the mechanism-based sufficiency account rather than the covariation approach. Experiment 2 replicated the finding of preferring sufficient joint attributions in Experiment 1.

Experiment 2 did not replicate the contrast effects found in the previous studies (Beller et al., 2009; Bender and Beller, 2011; Beller and Bender, 2015). However, the present finding that the majority of participants made joint attributions is consistent with the previous finding that the majority (53%) of participants gave relational or joint explanations for effects in Beller and Bender’s Experiment 2 (Beller and Bender, 2015). This suggests that the present response format is also valid for measuring causal attributions, like the causal explanation question used by them.

## General Discussion

Experiment 1 found that for a compound causation with explicit mechanism information, participants tended to attribute an effect to the joint influence of two agents on a patient. Experiment 2 found that for a simple causation without explicit mechanism information, participants tended to attribute an effect to the joint influence of an agent and a patient. Both experiments demonstrated that in both the presence and absence of contrast situations, participants generally preferred sufficient joint attributions over the other attributions regardless of whether mechanism information was present, and contrast situations did not affect causal attributions. Thus, sufficient joint attributions for compound causation with explicit mechanism information can generalize to simple causation without explicit mechanism information. To my knowledge, these findings are novel on the whole.

Overall, these findings favor the mechanism-based sufficiency account rather than the covariation approach and the complexity account. The mechanism-based sufficiency account predicts that people should attribute an effect to the joint influence of multiple relevant factors involved in the mechanism. The covariation approach predicts that a contrast situation should elicit contrast-factor attributions based on covariation involved in the contrast situation.

In Experiment 1, the predominance of sufficient joint attributions implies that when mechanism and covariation information are both present, sufficient joint attributions based on mechanism information are more sufficient for effects than contrast-factor attributions based on covariation information are. Thus, the influence of mechanism information trumps the influence of covariation information. Moreover, the predominance of sufficient joint attributions also implies that people prefer sufficient to over-sufficient joint attributions although over-sufficient joint attributions involve more information and are more complex than sufficient joint attributions. This suggests that preferences for complex joint attributions are modulated by explanatory sufficiency and are not directly related to explanatory complexity or amount of information. Thus, preferring joint attributions is not based on explanatory complexity or amount of information. This supports the mechanism-based sufficiency account, but not the complexity account.

Experiment 2 showed the predominance of joint attributions for simple causation in contrast situations without explicit mechanism information. This implies that people still prefer joint attributions to contrast-factor attributions even if explicit concrete mechanism information is absent. We explain this phenomenon as follows. When explicit concrete mechanisms are absent, people can make joint attributions by appealing to implicit abstract mechanism knowledge such as the general principle that an effect is generally brought about by the joint influence of multiple relevant factors involved in the causal mechanism. Such implicit abstract mechanism knowledge belongs to intuitive theories proposed by Gerstenberg and Tenenbaum (2017). For example, although people do not know the concrete mechanism of how a stone and a piece of glass interact to result in the piece of glass breaking, they know that the piece of glass breaking is due to the interaction of the two objects. Thus, the joint influence of the two objects is more sufficient than the influence of one object is. Thus, the implicit abstract mechanism knowledge can explain preferring joint attributions given no explicit mechanism information.

Experiment 2 found that symmetrical versus asymmetrical settings for simple causation did not affect causal attributions. Even though agents and patients play unequal roles in causation, participants still preferred joint attributions to contrast-factor attributions. This implies that in Experiment 2, the absence of contrast effects is not due to the setting type of causal relationships. This finding is different from the previous finding of contrast effects in physically symmetrical settings (Beller et al., 2009; Bender and Beller, 2011; Beller and Bender, 2015).

Overall, in both the presence and absence of contrast situations, people prefer sufficient joint attributions based on mechanism knowledge to contrast-factor attributions based on covariation information regardless of whether mechanism information is explicit. Causal attributions tend to rely on mechanism knowledge rather than covariation information, as predicted by the mechanism-based sufficiency account. This tendency can be explained as follows: causal mechanism involving multiple relevant factors is intrinsic to causation, whereas contrastive situations are extrinsic to causation. For an effect, the joint attribution implies a sufficient endogenous explanation, whereas the contrast-factor attribution implies an insufficient exogenous explanation with reference to external contrastive situations. The former is more sufficient than the latter. Thus, people tend to base their causal attributions on intrinsic mechanisms, showing the preference for joint attributions.

The predominance of sufficient joint attributions implies that people think that it is the joint of multiple relevant factors, rather than a single contrast factor, that brings about an effect. This finding resonates with the recent finding that in the explanation evaluation task, people have a preference for complex explanations that invoke more causes to explain an effect (Zemla et al., 2017). Preferring sufficient joint attributions and explanations is consistent with the mechanism approach. For example, classical mechanics argues that the motion of a body is determined by the resultant of forces acting on it. They are different from one-sided contrast-factor attributions that are prevalent in previous research on casual reasoning (Heider, 1958; Kelley, 1973; Einhorn and Hogarth, 1986; Mackie, 1986; Cheng and Novick, 1990, 1992; McGill and Klein, 1993; Cheng, 1997; Perales and Shanks, 2007; Wolff, 2007, 2017; Alicke et al., 2015; Hilton, 2017). To our knowledge, we for the first time demonstrate that people prefer sufficient joint explanations to one-sided contrast-factor explanations in contrast situations. This preference conforms to the idea that people engage in a process called “inference to the best explanation” (Lombrozo and Vasilyeva, 2017). That is, one infers that a hypothesis is likely to be true based on the fact that it best explains the data. In contrast situations, compared with the simple contrast-factor attribution, the joint attribution is sufficient for an effect, and thereby is the better explanation preferred by people.

The present finding of preferring sufficient attributions suggests that causal attributions are directly related to explanatory sufficiency, but not explanatory complexity. The influence of explanatory complexity is modulated by explanatory sufficiency. This is confirmed by the results of Experiment 1. The present finding of preferring sufficient attributions differs from the previous finding of preferring simpler explanations in the explanation evaluation task (Lombrozo, 2007; Pacer and Lombrozo, 2017; Johnson et al., 2018). Johnson et al. proposed the opponent heuristic account for the opponent preference pattern (Johnson et al., 2018). The idea is that objective simplicity/complexity of causal phenomena affects people’s preferences and people use the simplicity and complexity heuristic to explain simple and complex phenomena, respectively. The mechanism-based sufficiency account can explain this flexibility as follows. For a complex phenomenon, a complex explanation is sufficient, whereas a simple explanation is insufficient. In this case, people will prefer the complex explanation. For a simple phenomenon, a simple explanation is sufficient, whereas a complex explanation is unnecessary. In this case, people will prefer the simple explanation. When the simple explanation is sufficient for the effect, the complex explanation may artificially complicate matters and thereby is biased. Here, explanatory sufficiency is the primary factor that modulates people’s preferences for simplicity or complexity. Thus, preferences for simplicity or complexity are modulated by explanatory sufficiency, and thereby are flexible. This is confirmed by the present finding of preferring sufficient attributions.

The above analyses suggest that people’s preferences for simplicity/complexity may vary with some modulating variables, and thereby are flexible. These modulating variables include complexity of causal phenomena and explanatory sufficiency. The mechanism-based sufficiency account can integrate these variables together. How do these variables interact to affect causal attributions and explanations? This question is worth further study.

It is noteworthy that the mechanism-based sufficiency account does not equate to the conventional mechanism approach, though the mechanism-based sufficiency account and the mechanism approach have the same prediction for causal attributions in the present experiments. Specifically, the mechanism-based sufficiency account involves explanatory sufficiency that modulates the assessment of whether mechanism information is sufficient for an effect. However, explanatory sufficiency is not necessary for the mechanism approach, because there are some mechanism explanations that are partial and insufficient. For example, for the question about the glass breaking, the stone (the constant factor) is one factor involved in the mechanism, but not sufficient for the glass breaking. Thus, people do not take the constant-factor attribution based on one aspect of the mechanism. This is confirmed by the results of the present experiments.

The previous finding of contrast-factor attributions is essentially a pragmatic effect that resulted from the influence of covariation in contrast situations without mechanism information (Beller and Bender, 2015). Hilton (1990, 2017) demonstrated that some other pragmatic factors such as interpersonal explanation processes and variations in implicit contrasts can affect causal attributions and explanations. Thus, pragmatic factors such as contrast settings and conversational contexts can elicit contrast-factor explanations. Although our Experiment 2 did not find contrast effects, we conjecture that in a contrast situation, the way of questioning may affect whether contrast effects occur. In particular, if a question explicitly focuses on the difference between two contrastive instances, it will elicit contrast-factor explanations. For example, for the pair of “a stone does not break a steel plate” and “the stone breaks a piece of glass,” why is it the piece of glass, rather than the steel plate, that breaks? This contrastive question will elicit the contrast-factor explanation: the piece of glass breaking is because the piece of glass is more brittle than the steel plate. Our Experiment 2 used the non-contrastive question without focusing on the difference between contrastive instances, and thereby failed to find the contrast effect. Thus, contrast-factor attributions may result from contrastive questions, and thereby are pragmatic phenomena. This is consistent with the explaining difference view that regards attributions as explaining differences between different causal situations (Hesslow, 1983).

When using non-contrastive questions, the influence of mechanism knowledge may trump the influence of contrast settings, and people may prefer sufficient joint explanations to simple contrast-factor explanations, resulting in that contrast situations no longer affect causal attributions. This is confirmed by the results of Experiment 2. Thus, contrast situations are not bound to elicit contrast-factor attributions. Overall, for simple causation involving an agent and a patient, whether contrast situations can elicit contrast-factor attributions may depend on the way of questioning (contrastive versus non-contrastive questioning). Future studies should investigate this direction.

However, Experiment 1 revealed that given explicit mechanism information for compound causation involving two agents and a patient, the contrastive questions failed to elicit contrast-factor attributions, and participants still preferred joint attributions. This result suggests that given explicit mechanism information, even for contrastive questions, the influence of mechanism knowledge can trump the influence of contrast settings.

Moreover, the response format of questions can affect causal attributions. Experiment 2 failed to find contrast effects in contrast situations without explicit mechanism information. This result is different from the previous finding of contrast effects (Beller et al., 2009; Bender and Beller, 2011; Beller and Bender, 2015). This difference arises from the response format difference between our and their experiments. Our experiments used the multiple-option format that involves the joint attribution, whereas their experiments used the two-option format that involves only single-factor attributions. This difference implies that when the joint option is available, people generally prefer sufficient joint attributions to simple contrast-factor attributions regardless of whether there are contrast situations. Contrast situations fail to elicit contrast-factor attributions even if explicit mechanism information is absent. Thus, the contrast effects found in their studies resulted from the demand limitation of the two options used in Beller et al.’s studies. The dichotomous response format limited their participants’ choices to the two one-sided options and induced contrast-factor attributions. Such response format resulted in biased dichotomous thinking without considering the complex joint attribution. When not using the biased dichotomous response format, contrast effects will no longer occur. This is confirmed by the results of Experiment 2. Moreover, the present finding of the predominance of joint attributions is consistent with the previous finding that the majority (53%) of participants gave the relational or joint explanation for the effect in Beller and Bender’s Experiment 2 (Beller and Bender, 2015). This suggests that the present response format is valid, like the open explanation question used by them. Finally, we used more diverse casual contents (six kinds of causal contents) than they did (two kinds of physical causal contents: an object floating on a liquid and a gas staying down in a substance), and thereby the present findings were more generalizable. Overall, the present finding is more valid than their finding.

Overall, the aforementioned factors may affect whether people take sufficient joint attributions or other attributions. How do these factors affect people’s preferences in causal attribution? The mechanism-based sufficiency account promises to answer this question, because it integrates mechanism information and the sufficiency standard of causal attributions, and thereby seems to be able to accommodate the main modulating variables. Future studies should explore this possibility.

## Data Availability

Publicly available datasets were analyzed in this study. The datasets for this study can be found at the URL: http://dx.doi.org/10.17605/OSF.IO/P4SWX.

## Ethics Statement

This study was carried out in accordance with the recommendations of “Shaanxi Normal University Human Research Ethics Committee” with written informed consent from all subjects in accordance with the Declaration of Helsinki. The protocol was approved by the “Shaanxi Normal University Human Research Ethics Committee”.

## Author Contributions

MW conceived the research concept and completed writing. MZ performed the experiments and the statistical analyses.

### Conflict of Interest Statement

The authors declare that the research was conducted in the absence of any commercial or financial relationships that could be construed as a potential conflict of interest.

## References

[ref1] Ahn W. Bailenson J. (1996). Causal attribution as a search for underlying mechanisms: an explanation of the conjunction fallacy and the discounting principle. Cogn. Psychol. 31, 82–123. 8812022 10.1006/cogp.1996.0013

[ref2] Ahn W.-K. Kalish C. W. Medin D. L. Gelman S. A. (1995). The role of covariation versus mechanism information in causal attribution. Cognition 54, 299–352. 10.1016/0010-0277(94)00640-7, PMID: 7720361

[ref3] Alicke M. D. Mandel D. R. Hilton D. J. Gerstenberg T. Lagnado D. A. (2015). Causal conceptions in social explanation and moral evaluation: a historical tour. Perspect. Psychol. Sci. 10, 790–812. 10.1177/1745691615601888, PMID: 26581736

[ref4] Beller S. Bender A. (2015). How contrast situations affect the assignment of causality in symmetric physical settings. Front. Psychol. 5:1497. 10.3389/fpsyg.2014.01497, PMID: 25620937 PMC4287057

[ref5] Beller S. Bender A. Song J. (2009). Weighing up physical causes: effects of culture, linguistic cues and content. J. Cogn. Cult. 9, 347–365. 10.1163/156770909X12518536414493

[ref6] Bender A. Beller S. (2011). Causal asymmetry across cultures: assigning causal roles in symmetric physical settings. Front. Psychol. 2:231. 10.3389/fpsyg.2011.00231, PMID: 21960982 PMC3178231

[ref7] Bramley N. R. Gerstenberg T. Tenenbaum J. B. Gureckis T. M. (2018). Intuitive experimentation in the physical world. Cogn. Psychol. 105, 9–38. 10.1016/j.cogpsych.2018.05.001 29885534

[ref8] Buehner M. J. (2005). Contiguity and covariation in human causal inference. Learn. Behav. 33, 230–238. 10.3758/BF03196065 16075841

[ref9] Cheng P. W. (1997). From covariation to causation: a causal power theory. Psychol. Rev. 104, 367–405. 10.1037/0033-295X.104.2.367

[ref10] Cheng P. W. Novick L. R. (1990). A probabilistic contrast model of causal induction. J. Pers. Soc. Psychol. 58, 545–567. 10.1037/0022-3514.58.4.545, PMID: 2348358

[ref11] Cheng P. W. Novick L. R. (1992). Covariation in natural causal induction. Psychol. Rev. 99, 365–382. 10.1037/0033-295X.99.2.365, PMID: 1594730

[ref12] Craver C. F. Tabery J. (2015). “Mechanisms in science” Stanford Encyclopedia of Philosophy Center for the Study of Language and Information (CSLI), Stanford University.

[ref13] Danks D. (2005). The supposed competition between theories of human causal inference. Philos. Psychol. 18, 259–272. 10.1080/09515080500169371

[ref14] Einhorn H. J. Hogarth R. M. (1986). Judging probable cause. Psychol. Bull. 99, 3–19. 10.1037/0033-2909.99.1.3

[ref16] Fugelsang J. A. Thompson V. A. (2000). Strategy selection in causal reasoning: when beliefs and covariation collide. Can. J. Exp. Psychol. 54, 15–32. 10.1037/h0087327, PMID: 10721236

[ref17] Gerstenberg T. Tenenbaum J. B. (2017). “Intuitive theories” in Oxford handbook of causal reasoning. ed. Waldmannn M. (Oxford, England, UK: Oxford University Press), 515–548.

[ref18] Glymour C. N. (2001). The mind’s arrows: Bayes nets and graphical causal models in psychology. Cambridge, Massachusetts, USA: MIT Press.

[ref19] Heider F. (1958). The psychology of interpersonal relations. New York: Wiley.

[ref20] Hesslow G. (1983). Explaining differences and weighting causes. Theoria 49, 87–111.

[ref21] Hilton D. J. (1990). Conversational processes and causal explanation. Psychol. Bull. 107, 65–81. 10.1037/0033-2909.107.1.65

[ref22] Hilton D. J. (2017). “Social attribution and explanation” in The Oxford handbook of causal reasoning. ed. Waldmann M. (Oxford, England, UK: Oxford University Press).

[ref23] Illari P. M. Williamson J. (2012). What is a mechanism? Thinking about mechanisms across the sciences. Eur. J. Philos. Sci. 2, 119–135. 10.1007/s13194-011-0038-2

[ref24] Johnson S. G. Ahn W. K. (2017). “Causal mechanisms” in The Oxford handbook of causal reasoning. ed. Waldmann M. (Oxford, England, UK: Oxford University Press).

[ref25] Johnson S. Valenti J. J. Keil F. (2018). Simplicity and complexity preferences in causal explanation: An opponent heuristic account. 10.1016/j.cogpsych.2019.05.004 31200208

[ref26] Kelley H. H. (1973). The processes of causal attribution. Am. Psychol. 28, 107–128. 10.1037/h0034225

[ref27] Lake B. M. Ullman T. D. Tenenbaum J. B. Gershman S. J. (2017). Building machines that learn and think like people. Behav. Brain Sci. 40, 1–72. 10.1017/S0140525X16001837, PMID: 27881212

[ref28] Lombrozo T. (2007). Simplicity and probability in causal explanation. Cogn. Psychol. 55, 232–257. 10.1016/j.cogpsych.2006.09.006, PMID: 17097080

[ref29] Lombrozo T. Vasilyeva N. (2017). “Causal explanation” in Oxford handbook of causal reasoning. ed. Waldmann M. (Oxford, England, UK: Oxford University Press).

[ref30] Machamer P. Darden L. Craver C. F. (2000). Thinking about mechanisms. Philos. Sci. 67, 1–25. 10.1086/392759

[ref31] Mackie J. L. (1986). The cement of the universe. Oxford: Clarendon Press.

[ref34] McGill A. L. Klein J. G. (1993). Contrastive and counterfactual reasoning in causal judgment. J. Pers. Soc. Psychol. 64, 897–905. 10.1037/0022-3514.64.6.897

[ref35] Morris M. W. Peng K. (1994). Culture and cause: American and Chinese attributions for social and physical events. J. Pers. Soc. Psychol. 67:949.

[ref36] Pacer M. Lombrozo T. (2017). Ockham’s razor cuts to the root: simplicity in causal explanation. J. Exp. Psychol. Gen. 146, 1761–1780. 10.1037/xge0000318, PMID: 29251989

[ref37] Perales J. C. Shanks D. R. (2007). Models of covariation-based causal judgment: a review and synthesis. Psychon. Bull. Rev. 14, 577–596. 10.3758/BF03196807, PMID: 17972719

[ref38] White P. A. (1989). A theory of causal processing. Br. J. Psychol. 80, 431–454. 10.1111/j.2044-8295.1989.tb02334.x

[ref39] White P. A. (2009). Property transmission: an explanatory account of the role of similarity information in causal inference. Psychol. Bull. 135, 774–793. 10.1037/a0016970 19702382

[ref40] White P. A. (2012). The experience of force: the role of haptic experience of forces in visual perception of object motion and interactions, mental simulation, and motion-related judgments. Psychol. Bull. 138, 589–615. 10.1037/a0025587, PMID: 22730922

[ref41] White P. A. (2014). Singular clues to causality and their use in human causal judgment. Cogn. Sci. 38, 38–75. 10.1111/cogs.12075, PMID: 23957568

[ref42] Wolff P. (2007). Representing causation. J. Exp. Psychol. Gen. 136, 82–111. 10.1037/0096-3445.136.1.82, PMID: 17324086

[ref43] Wolff P. (2017). “Force dynamics” in Oxford handbook of causal reasoning. ed. Waldmann M. (Oxford, UK: Oxford University Press).

[ref44] Zemla J. C. Sloman S. Bechlivanidis C. Lagnado D. A. (2017). Evaluating everyday explanations. Psychon. Bull. Rev. 24, 1488–1500. 10.3758/s13423-017-1258-z, PMID: 28275989

